# Androgen Receptors Expression in Pituitary of Male Viscacha in relation to Growth and Reproductive Cycle

**DOI:** 10.1155/2015/168047

**Published:** 2015-04-07

**Authors:** Verónica Palmira Filippa, Gabriela Judith Rosales, Albana Andrea Marina Cruceño, Fabian Heber Mohamed

**Affiliations:** ^1^Histología, Facultad de Química, Bioquímica y Farmacia, Universidad Nacional de San Luis, Avenida Ejército de los Andes 950, Bloque I, Piso No. 1, 5700 San Luis, Argentina; ^2^Consejo Nacional de Investigaciones Científicas y Técnicas (CONICET), 5700 San Luis, Argentina

## Abstract

The aim of this work was to study the androgen receptors (AR) expression in pituitary pars distalis (PD) of male viscachas in relation to growth and reproductive cycle. AR were detected by immunocytochemistry and quantified by image analysis. Pituitary glands from fetus, immature, prepubertal, and adult viscachas during their reproductive cycle were used. In the fetal PD, the immunoreactivity (ir) was mainly cytoplasmic. In immature and prepubertal animals, AR-ir was cytoplasmic (ARc-ir) and nuclear (ARn-ir) in medial region. In adult animals, ARn-ir cells were numerous at caudal end. AR regionalization varied between the PD zones in relation to growth. In immature animals, the ARn-ir increased whereas the cytoplasmic expression decreased in relation to the fetal glands. The percentage of ARc-ir cells increased in prepubertal animals whereas the nuclear AR expression was predominant in adult viscachas. The AR expression changed in adults, showing minimum percentage in the gonadal regression period. The variation of nuclear AR expression was directly related with testosterone concentration. These results demonstrated variations in the immunostaining pattern, regionalization, and number of AR-ir cells throughout development, growth, and reproductive cycle, suggesting the involvement of AR in the regulation of the pituitary activity of male viscacha.

## 1. Introduction

Androgens are important steroidal hormones involved in the sexual development and reproduction of male mammals. They are essential during development and for the maintenance of secondary male characteristics, initiation and continuation of spermatogenesis, and gonadotropin regulation [1]. Testosterone and dihydrotestosterone bind to specific cytosolic receptors, the androgen receptors (AR), resulting in a conformational change in receptor protein, dimerization, nuclear translocation, association with cofactors, and interaction with specific regions of the genome [2] to initiate a cellular response [3]. The existence of the AR expression in the cytoplasm of pituitary cells has been described in other species [4, 5]. It has been demonstrated that there is an inverse relationship between the cytoplasmic and nuclear localization, and testosterone administration increases the nuclear expression of AR [6].

Several studies have demonstrated that the pituitary gland is an androgen target tissue and serves as a potential site of androgen feedback on hormone secretion [4, 6, 7]. The localization and function of AR have been studied in the pituitary gland in some mammalian species in relation to age or gender [8–10]. AR expression has been studied during the fetal development of the hypothalamus, hippocampus, and pituitary in monkeys [11, 12] and sheep [13]. Studies of the ontogeny of the AR expression in the developing pituitary gland have been performed in monkeys [14], chickens [15], mice [16], rats [17], and sheep [18], and it has been demonstrated that pituitary AR in mammals is conservative.

Some species use environmental cues, the most important being the photoperiod, to synchronize their endogenous cycles and to reproduce at the optimal time of the year [19]. The pituitary gland is under control of the hypothalamus and the gonadal hormones, which exert feedback effects on the hypothalamic-pituitary-gonadal axis [20]. Variations in the androgen serum concentration and their receptors in different tissues are responsible for biological effects throughout the life cycle of adult animals. Testosterone plays a highly significant role in the transition from the reproductive activity phase to gonadal regression and gonadal recrudescence [21]. Seasonal variations in testosterone serum concentration are related to the reproductive status of male birds [22] and hamsters [23]. The central mechanisms responsible for the photoperiod negative effects upon the testosterone and the possible influence of day length on AR expression have been reported [24]. Tetel et al. [25] have indicated that day-length-induced fluctuations in AR expression may contribute to seasonal variations of testosterone.

Our experimental model, the viscacha (*Lagostomus maximus maximus*), is a hystricomorph rodent of seasonal reproductive patterns. The annual reproductive cycle of adult male animals has three periods: reproductive (summer and early autumn), gonadal regression (winter), and gonadal recovery (spring). Previous studies have demonstrated that the gonadal regression period is characterized by an increase of the secretory ability of the pineal gland [26] and maximum levels of melatonin in blood [27]. In pituitary PD the activity of LH- and FSH-cells was different during the annual reproductive cycle, demonstrating that these cells do not secrete in parallel, and melatonin acts differentially on the gonadotroph activity [28]. In addition, corticotrophs, somatotrophs, thyrotrophs, and lactotrophs vary throughout the reproductive cycle [29–32]. A reduction of the testicular weight and of the seminiferous tubule diameters, seminiferous epithelium with Sertoli cells, spermatogonia and a few primary spermatocytes, and hypotrophic Leydig cells was observed in winter [33, 34]. The testicular activity was slowly recovered during spring, reaching its maximum during summer and early autumn in the reproductive period. More recent results from adult male viscachas have demonstrated seasonal changes in the structures of seminal vesicles according to the AR expression and testosterone serum concentrations [35]. Cruceño et al. [36] reported that the epididymal sperm morphology underwent changes during the reproductive cycle, with the highest quantity of abnormal gametes during the gonadal regression period.

Earlier histological studies have described different pituitary PD zones, called ventral, medial, and dorsal regions and rostral and caudal ends. It has been demonstrated that pituitary cell types changed in relation to age and gonadal activity of viscachas [29, 30, 32]. Changes in the immunostaining for AR and LH- and FSH-cells have been recently reported in the pituitary of castrated adult male viscachas. The immunocolocalization study showed that cells expressing AR and FSH were more affected by the lack of gonadal androgens, suggesting that some gonadotroph subpopulations may exist with different regulatory mechanisms for hormonal synthesis, storage, and secretion [37].

The expression of AR in the pituitary of fetal, immature, and prepubertal viscachas has not been examined yet, and there is no data about the seasonal expression of AR in the pituitary gland of this rodent. Based on the above data and our previous knowledge about the viscacha, it is reasonable to hypothesize that pituitary AR expression changes throughout development, growth, and reproductive cycle of male viscacha. To test this hypothesis, we performed an immunohistochemical and morphometrical study of AR in the pituitary PD of male viscachas, during development, growth, and the annual reproductive cycle. This immunohistochemical study might be useful for discussing the probable role of androgens in the pituitary development of viscachas and their participation in the reproductive stage along the year.

## 2. Materials and Methods

### 2.1. Animals

The viscachas were captured in their habitat near San Luis, Argentina (33° 20′ south latitude, 760 m altitude), using traps placed in their burrows. In San Luis, in summer, the light phase is up to 14 h light daily (14L : 10D) with an average temperature of 25°C. In winter, the light phase decreases to 10 h (10L : 14D), and the average temperature is 10°C. In spring, the light phase increases to 12 h (12L : 12D), and the average temperature is 15°C.

A total of eight fetal male pituitary glands were collected. The fetal pituitaries were collected from fetuses at mid- (*n* = 4) and late (*n* = 4) pregnant females classified on the basis of fetal weight and crown-heel length [38, 39]. The male animals were carefully classified into immature (1-2 kg; *n* = 4) and prepubertal (3-4 kg; *n* = 4) according to body weight and light microscopy observations of testes [30, 40]. Twelve adult male viscachas weighing 5–7 kg were captured during the most representative months of their reproductive cycle, for a period of over 1 year: 4 animals during the reproductive period in summer to early autumn (February to April), 4 animals in the gonadal regression period in winter (July), and 4 animals in the gonadal recovery period in spring (September). The reproductive condition of viscachas was carefully assessed on the basis of observations by light microscopy of testes.

The animals were intramuscularly anesthetized with a combination of ketamine (Ketamina 50, Holliday-Scott SA) and xylazine (Vetanarcol, König SA) at a dose of 12 and 0.4 mg/kg, respectively. The blood was collected for cardiac puncture for the evaluation of serum hormone concentration and quickly sacrificed by intracardiac injection of Euthanyle (0.25 mL/Kg body weight, sodium pentobarbital, sodium diphenylhydantoin, Brouwer S.A.). The brain was rapidly exposed and the pituitary processed for light microscopy. The pituitaries were fixed in Bouin's fluid, embedded in paraffin, and sagittally sectioned (5 *μ*m thick), following the design used in previously reported studies [30, 32].

The experimental design was approved by the local ethics committee and was in agreement with the National Institute of Health (NIH, USA) guidelines for the use of experimental animals. Moreover, the Biodiversity Control Area of the San Luis Ministry of the Environment (Argentina) approved a study protocol for conducting scientific research within the territory of this province (Resolution number 45.PBD.2014).

### 2.2. Immunohistochemistry of AR in the Pituitary

**Figure 1:**
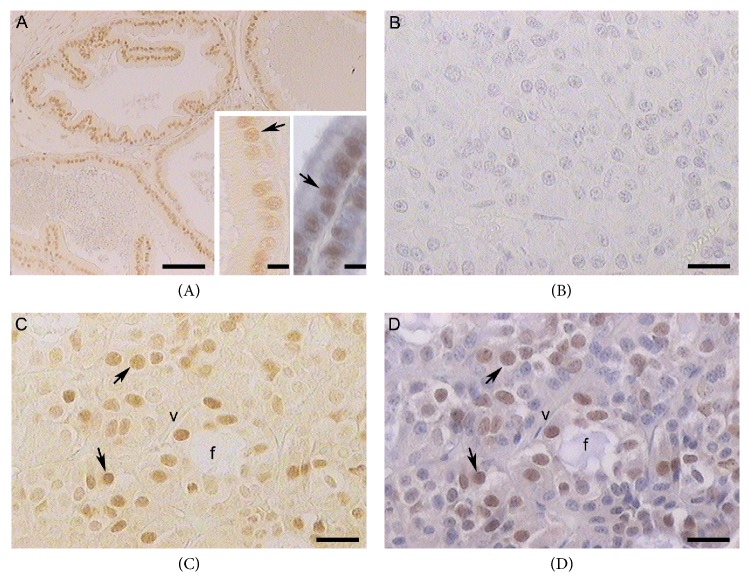
(A) Rat prostate, a positive control of immunoperoxidase staining with anti-AR (N-20) without hematoxylin staining. Insets: immunolabeled nuclei of epithelial cells (arrows) without (left) and with (right) counterstained hematoxylin. (B) Pituitary of viscacha, a negative control of immunohistochemistry counterstaining with hematoxylin. ((C), (D)) Pituitary of viscacha, staining with anti-AR (N-20) without hematoxylin staining (C) and counterstaining with hematoxylin (D). ARn-ir cells (arrows). Blood vessels, v; follicular structures, f. (A) Scale bar: 100 *μ*m. (B), (C), and (D) and* insets* scale bar: 25 *μ*m.

The tissue sections were stained using the streptavidin-biotin peroxidase complex method at 20°C. The sections were first deparaffinized with xylene, hydrated through decreasing concentrations of ethanol, and rinsed with distilled water and phosphate-buffered saline (PBS, 0.01 M, pH 7.4). Antigen retrieval was performed by microwaving the sections for 6 min (2 × 3 min) at full power in a sodium citrate buffer (0.01 M, pH 6.0). Endogenous peroxidase activity was inhibited with 3% H_2_O_2_ in water for 20 min. Nonspecific binding sites for immunoglobulins were blocked by incubation for 20 min with normal serum diluted in PBS containing 1% bovine serum albumin, 0.09% sodium azide, and 0.1% Tween-20. Sections were incubated with the primary antibody: 6 hours in a humidified chamber at 20°C with rabbit polyclonal anti-human (h) AR (N-20; Santa Cruz Biotechnology, Santa Cruz, CA, USA). After rinsing with PBS for 10 min, immunohistochemical visualization was carried out using the Super Sensitive Ready-to-Use Immunostaining Kit (BioGenex, San Ramon, CA, USA), which was used as follows: sections were incubated for 30 min with diluted biotinylated anti-IgG and, after being washed in PBS, were incubated for 30 min with horseradish peroxidase conjugated streptavidin and were finally washed in PBS. AR immunoreactivity cells (AR-ir cells) were visualized using freshly prepared solution with 100 *μ*L 3,3′-diaminobenzidine tetrahydrochloride chromogen in 2.5 mL PBS and 50 *μ*L H_2_O_2_ substrate solution. In all cases, two experiments for controlling the specificity of the primary antibody were made: (i) omission of primary antibody and (ii) adsorption of primary antibody with a homologous antigen. No positive structures or cells were found in these sections. Rat prostate was used as positive control (Figure 1).

### 2.3. Morphometric Analysis

A computer-assisted image analysis system was used for the morphometric analysis as previously reported [37, 41]. The image was displayed on a color monitor; a standard area of 18141.82 *μ*m^2^ (reference area) was defined on the monitor; distance calibration was performed using a slide with a micrometric scale for microscopy (Reichert, Austria). The morphometric study was carried out as follows: 3-4 regularly spaced serial tissue sections (100 *μ*m each) from a pituitary were used, and microscopic fields were examined under a 40x objective. In each section, 25 microscopic fields were randomly selected throughout the PD, five from each region (ventral, medial, and dorsal regions) or end (rostral and caudal ends) of the PD. In each image, the percentage of immunoreactive (-ir) cells in the PD (percentages of ARn-ir, ARc-ir) was obtained according to the formula *A*/(*A* + *B*) × 100. Each image contained approximately 250–280 cells. The number of immunoreactive cells (*A*) and the number of nuclei in immunonegative cells (*B*) were counted.

### 2.4. Testosterone Serum Concentrations

Blood samples were incubated at 4°C for 30 min and centrifuged at 5,000 g for 5 min, and serum was removed. Testosterone serum concentrations were measured using the total testosterone test as previously reported by Chaves et al. [35]. This test is a solid-phase competitive chemiluminescent enzyme immunoassay run on a Siemens^∗^ Medical IMMULITE^∗^ 1000 Immunoassay Analyzer (Siemens Medical Solutions Diagnostics).

### 2.5. Statistical Analysis

The results were expressed as mean ± the standard error of the mean (SEM) for all morphological data sets. The data were analyzed by nonparametric test procedure, the Kruskal-Wallis test, for overall significance of group, followed by the Mann-Whitney test for individual comparisons, using the Infostat 2008 version. A *P* value of less than 0.05 was considered statistically significant.

## 3. Results

AR-ir cells were studied by immunohistochemistry on each zone of the pituitary PD parenchyma of fetal, immature, prepubertal, and adult male viscachas. The AR-ir cells were observed throughout the PD parenchyma, and immunohistochemical results revealed their presence in the nuclei (ARn-ir) and in the cytoplasm (ARc-ir).

**Figure 2:**
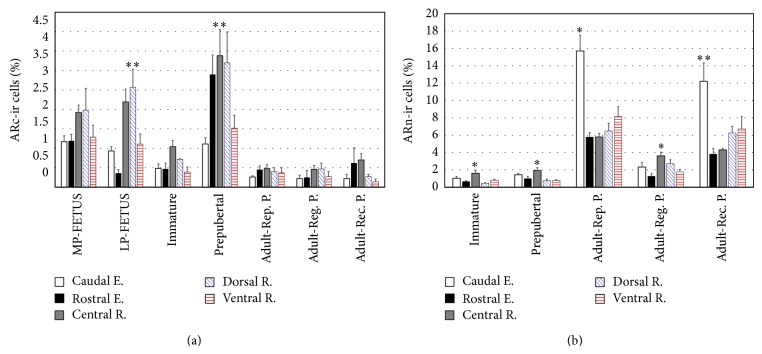
Morphometry of AR-ir cells in pituitary of male viscacha in different pituitary PD zones. (a) % ARc-ir cells. LP-FETUS: ^∗∗^ *P* < 0.01 dorsal R. versus ventral R., rostral E., and caudal E. Prepubertal: ^∗∗^ *P* < 0.01 medial R. versus ventral R. and caudal E. Adults: the percentage of ARc-ir cells did not reveal statistically significant changes in PD zones. (b) % ARn-ir cells. Immature: ^∗^ *P* < 0.05 medial R. versus dorsal R. and rostral E. Prepubertal: ^∗^ *P* < 0.05 medial R. versus dorsal R. and ventral R. Adult-Rep. P.: ^∗^ *P* < 0.05 caudal E. versus the other PD zones. Adult-Reg. P.: ^∗^ *P* < 0.05 medial R. versus ventral R. and rostral E. Adult-Rec. P.: ^∗∗^ *P* < 0.01 caudal E. versus rostral E. and medial R. Fetuses from midpregnancy, MP-FETUS; fetuses from late pregnancy, LP-FETUS; reproductive period, Rep. P.; gonadal regression period, Reg. P.; gonadal recovery period, Rec. P.; region, R.; end, E.

**Figure 3:**
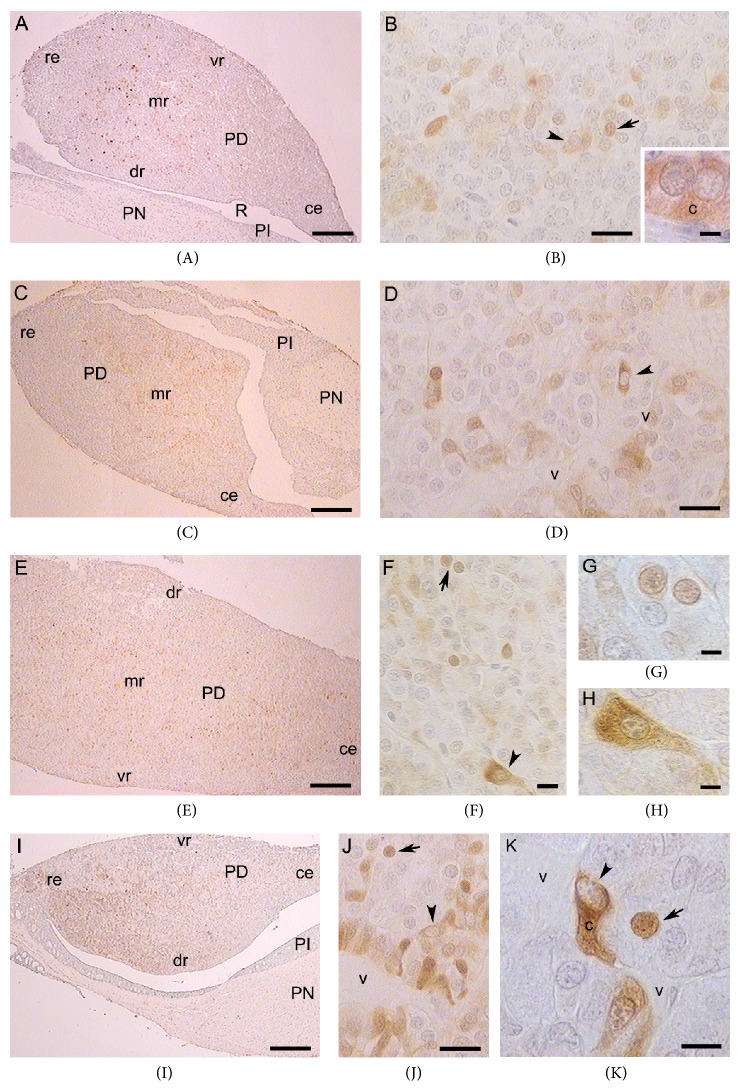
AR immunohistochemical staining of viscacha pituitaries. ((A), (B)) Sections of a fetal pituitary from midpregnancy showing the different zones of PD (A). In the medial region ARn-ir and ARc-ir cells were present (B).* Inset:* ARc-ir cells (c) with a negative nucleus. ((C), (D)) Images of a fetal pituitary from late pregnancy. Isolated ARc-ir cells around blood vessel (D). ((E), (F), (G), and (H)) AR-ir cells in a pituitary gland of immature viscacha. There are cells throughout the PD (E). The ARn-ir cells are increased in the medial region (F). Magnification of ARn-ir cells (G) and a pituitary cell with heterogeneous cytoplasmic staining (H). ((I), (J), and (K)) Pituitary of a prepubertal viscacha. ARc-ir cells in groups in contact with blood vessel (J) and others with a cytoplasmic prolongation (K). ARn-ir cells (arrows), ARc-ir cells (arrowheads). PD, pars distalis; PI, pars intermedia; PN, pars nervosa; R, Rathke's pouch; ce, caudal end; re, rostral end; vr, ventral region; mr, medial region; dr, dorsal region; blood vessels, v. ((A), (C), and (I)) Scale bar 500 *μ*m. (E) Scale bar 250 *μ*m. ((B), (D), (F), and (J)) Scale bar 25 *μ*m.* Inset of* (B), (G), (H), and (K) scale bar 10 *μ*m. Images counterstaining with hematoxylin.

In fetal pituitary from midpregnancy, scarce ARn-ir cells and intensely stained ARc-ir cells were located in the medial region and extended towards the ventral and dorsal regions. However, the percentages of ARn-ir cells (caudal end: 0.04 ± 0.02%; rostral end: 0.05 ± 0.03%; central region: 0.55 ± 0.15; dorsal region: 0.15 ± 0.07%; ventral region: 0.16 ± 0.07%) and the percentages of ARc-ir cells did not differ significantly among different zones of the PD (*P* > 0.05) (Figures 2(a), 3(A), and 3(B)).

In fetal pituitary from late pregnancy, ARc-ir cells were found around blood vessels or among other pituitary parenchymal cells. The percentage of ARc-ir cells in the dorsal region was significantly higher (*P* < 0.01) than in the ventral region and at the rostral and caudal ends (Figures 2(a), 3(C), and 3(D)). ARn-ir cells were few and frequently spherical, and some of them were irregular in shape. The percentage of these cells did not vary among different PD zones (*P* > 0.05; caudal end: 0.54 ± 0.23%; rostral end: 0.21 ± 0.02%; central region: 0.25 ± 0.10; dorsal region: 0.40 ± 0.10%; ventral region: 0.28 ± 0.10%).

In pituitary PD of immature animals, the ARc-ir cells were located in the medial region and extended towards the caudal end. The cytoplasmic immunostaining was less intense than in fetal glands cells. Several ARc-ir cells were oval and their spherical nucleus was located at one end of the cytoplasm. The percentage of ARc-ir cells did not differ significantly (*P* > 0.05) in different PD zones (Figure 2(a)). Intensely stained ARn-ir cells were mainly observed in the medial region, where their percentage was significantly higher than in the dorsal region and rostral end (*P* < 0.05; Figures 2(b) and 3(E)–3(H)).

In prepubertal viscachas, numerous immunopositive AR-ir cells were distributed throughout the PD parenchyma. These cells were arranged around blood vessels at the rostral end and in the medial and dorsal regions. In the ventral region, there were some irregular AR-ir cells with a cytoplasmic prolongation surrounding blood vessels or between other parenchymal cells. The ARc-ir cells showed significant variations; the highest value was found in the medial region in relation to the ventral region and caudal end (*P* < 0.01) whereas the lowest percentage was found at the caudal end (Figure 2(a)). The immunostaining of ARn-ir cells was intense in the dorsal region, and the highest percentage of these cells was found in the medial region in relation to the dorsal and ventral regions and rostral end (*P* < 0.05; Figures 2(b) and 3(I)–3(K)).

**Figure 4:**
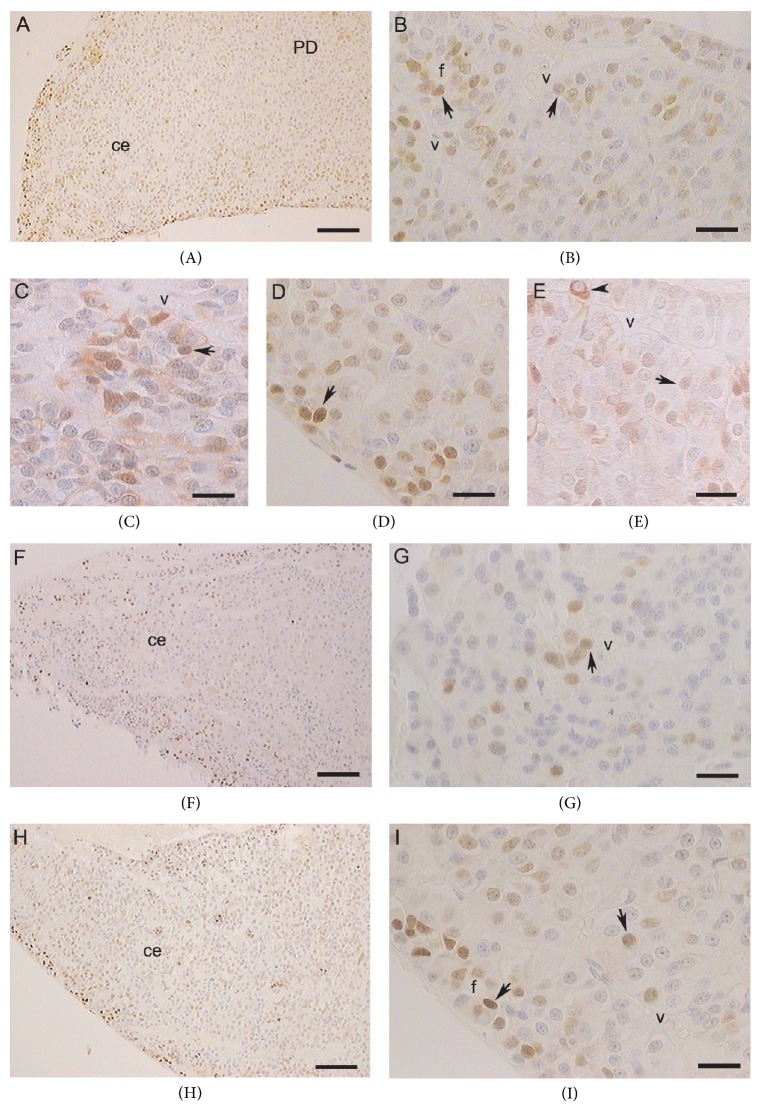
Seasonal expression of AR in pituitary during the reproductive cycle of adult viscachas. ((A)–(E)) Reproductive period. ((A), (B)) Images of caudal end; the AR were expressed mainly in the nuclei of PD cells (arrows). ((C), (D), and (E)) AR expression at the rostral end, ventral region, and dorsal region, respectively. The arrowhead indicated a cell with cytoplasmic labeling for AR. ((F), (G)) A few AR-ir cells at caudal end during the gonadal regression period. ((H), (I)) AR-ir cells at caudal end during the gonadal recovery period. Caudal end, ce; blood vessels, v; follicular structures, f. ((A), (F), and (H)) Scale bar 100 *μ*m. ((B), (C), (D), (E), (G), and (I)) Scale bar 25 *μ*m. Images counterstaining with hematoxylin.

In PD of adult animals, the AR expression mainly had a nuclear localization. In the reproductive period, several positive nuclei were found at the caudal end and dorsal region as well as on the dorsal and ventral edges of PD. The percentage of ARn-ir cells was significantly higher at the caudal end in relation to the other PD zones (*P* < 0.05; Figure 2(b)). A small number of ARc-ir cells were frequently located in the medial region. The cytoplasmic immunolabeling pattern was homogeneous and sometimes intense. In addition, some cells presented heterogeneous cytoplasmic staining and positivity on the nuclear periphery (Figures 4(A)–4(E)). No significant changes were observed in the percentage of ARc-ir cells between different PD zones (*P* > 0.05; Figure 2(a)).

In the gonadal regression period, AR-ir cells were mainly found in the medial region of PD. The immunostained cytoplasms were small, with medial or eccentric nucleus. There were no significant differences in ARc-ir cells between different PD zones (*P* > 0.05; Figure 2(a)), whereas ARn-ir cells were more numerous in the medial region in relation to the ventral region and the rostral end (*P* < 0.05; Figure 2(b)).

In the gonadal recovery period, an increase of AR-ir cells throughout PD parenchyma was observed. In this period, the cytoplasmic labeling was more intense and homogeneous in relation to the gonadal regression period (Figures 4(F)–4(I)). The percentage of ARc-ir cells was not significantly different between the PD zones (*P* > 0.05; Figure 2(a)). The percentage of ARn-ir cells was higher at the caudal end in relation to the rostral end and the medial region (*P* < 0.01; Figure 2(b)).  Table 1 shows a summary of the main cytological characteristics.

**Figure 5:**
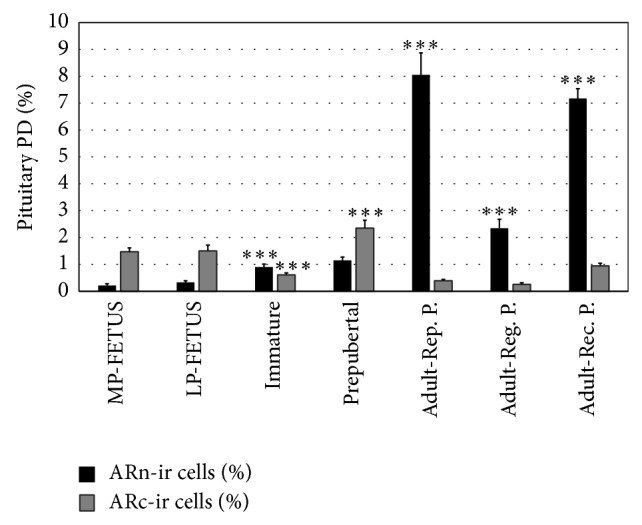
Total percentage of ARn-ir cells and ARc-ir cells in fetus (from mid- and late pregnancy), immature, prepubertal, and adult viscachas (during reproductive, gonadal regression, and gonadal recovery periods). The result revealed statistically significant changes: ^∗∗∗^ *P* < 0.001 immature versus LP-FETUS; prepubertal versus immature; Adult-Rep. P. versus prepubertal; Adult-Reg. P. versus Adult-Rep. P.; Adult-Rec. P. versus Adult-Reg. P.

The morphometric variations of the AR expression in relation to development and growth in viscachas PD were studied by comparing the data obtained from fetal pituitary during mid- and late pregnancy and from immature, prepubertal, and adult pituitary in the reproductive period. There was an increase in ARn-ir cells percentage and a significant decrease in the percentage of ARc-ir cells in immature animals as compared to fetus and in adult animals during the reproductive period as compared to prepubertal animals. The percentage of ARc-ir cells increased significantly in prepubertal in relation to immature viscachas. Statistical analysis is shown in Figure 5.

In order to study the seasonal expression of AR, the three periods of the reproductive cycle were analyzed. The total percentages of ARn-ir and ARc-ir cells obtained from three periods of the reproductive cycle of adult male viscachas were statistically analyzed (Figure 5). The percentage of ARn-ir cells showed a significant decrease during the gonadal regression period (*P* < 0.001). No variations were observed in the percentage of ARc-ir cells throughout the reproductive cycle (*P* > 0.05).

**Figure 6:**
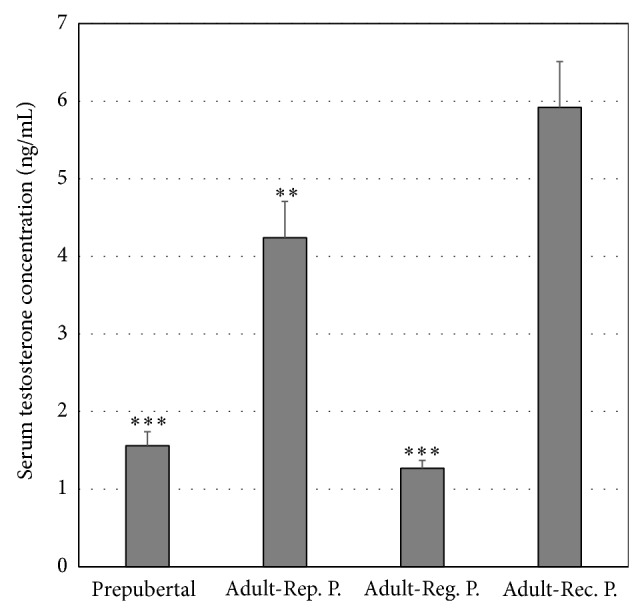
Testosterone serum concentrations. The values are expressed as mean ± SEM (*n* = 4). ^∗∗^ *P* < 0.01: Adult-Rep. P. versus Adult-Reg. P.; ^∗∗∗^ *P* < 0.001: prepubertal versus Adult-Rep. P.; Adult-Reg. P. versus Adult-Rec. P.

The testosterone serum concentrations of immature animals were below the detection limit (<0.20 ng/mL) and too low to reliably reveal differences from the other groups. The testosterone serum concentrations of prepubertal and adult viscachas in the gonadal regression period were lower than those in adult viscachas during their reproductive and gonadal recovery periods (*P* < 0.001; Figure 6).

## 4. Discussion

The analysis of the AR expression in the viscacha pituitary gland is essential for a better understanding of the regulatory systems since androgens are involved in the control of the reproductive function. This study represents the first description of variations in the regionalization and in the immunostaining pattern of pituitary AR in relation to the development, growth, and reproductive cycle of male viscachas.

The main findings of this study can be summarized as follows: (1) the regionalization and immunostaining pattern of pituitary AR vary in relation to growth; (2) the AR expression was mainly cytoplasmic (ARc-ir) in fetuses; (3) a significant increase of ARc-ir cells was observed in prepubertal animals; (4) the nuclear AR (ARn-ir) expression increased in relation to development, reaching the highest values in adult animals; (5) the ARn-ir cells varied seasonally with maximum values during the reproductive period and minimum values during the gonadal regression period; (6) the amount of ARn-ir cells was directly related to testosterone serum concentration.

There are still many questions about the relations of AR expression with the pituitary development and hormone secretion. Wu et al. [12] did not observe AR in the pituitary gland during late gestation and suggested that the lack of feedback by way of AR at pituitary levels may have important consequences on the fetal pituitary function. More recently, Yuan et al. [18] have reported in sheep that AR emerges in the anterior pituitary before day 60 of gestation, and the percentage of the total anterior pituitary cells expressing the receptor did not change throughout gestation. In addition, other studies have concluded that the fetal brain and pituitary are target sites for circulating androgens or androgen precursors in fetal plasma, and the increase of androgens in hypothalamic action immediately prior to birth might be critical to the timing of parturition [13]. Huang and Yuan [42] have reported that AR expression reflects the functional status of androgen in the pituitary of male sheep lamb. These authors suggest that during sexual maturity the feedback of sex hormones on the pituitary is at low level and stimulates gonadotropin secretion.

Some reproductive characteristics of female viscachas, such as polyovulation, implantation, and offspring birth, have been described in previous studies. Gestation lasts approximately 154 days, an unusually long period for a rodent, and it finishes in spring when the survival probabilities are ideal for the mother and offspring [38, 43, 44]. The results obtained in this work demonstrate AR in fetal pituitary from midpregnancy, indicating that AR are present around days 75–80 of gestation in the pituitary cells. These receptors are potentially in position to participate in the regulation of the fetal androgen function. Furthermore, the analysis of the distribution, morphometry, and immunostaining pattern of AR-ir cells in the parenchyma of fetal glands suggests that these cells contain variable amounts of AR, and they are morphologically related to blood supply and other endocrine cells of PD parenchyma. In late pregnancy of viscacha, the percentages of ARc-ir and ARn-ir cells did not change with respect to the values found during midpregnancy. These results suggested that fetal pituitary AR might have any specific function since midgestation in viscacha.

The hypothalamic-pituitary-gonadal axis is activated during puberty when hormonal modulation of sexual behavior is organized [45]. During male development, the masculinization is given by the hormones produced by the fetal testes, primarily testosterone [46, 47]. The gonadal steroids feed back to the central nervous system to exert a key role in the central control of the reproductive axis by regulating the release of the gonadotropin-releasing hormone (GnRH) [48]. However, the neurons that synthesize GnRH did not express AR and there are sex-steroids sensitive neurons upstream the GnRH cells, where the feedback signals of the steroid might be processed to the GnRH cells [49]. The neurons of hypothalamic nuclei that expressed kisspeptin (K) and neurokinin B (NKB) may sense circulating levels of testosterone through the expression of AR [49, 50]. In rodents, sheep, and primates, K and NKB have recently been implicated in the control of puberty and the maintenance of the reproductive function in adulthood [51–53]. In the pituitary, the expression of AR has been mainly observed in gonadotrophs [18, 54, 55] and gonadal steroid hormones affect the gonadotroph population in order to regulate their activity.

In immature male viscachas, a significant increase of nuclear AR and an important decrease of cytoplasmic AR were observed. In prepubertal viscachas, more nuclei and cytoplasm appear, and there is a clear increase of ARc-ir cells mainly in the medial region, suggesting that more cells begin to express AR in preparation for sexual maturation. In adult, the immunostaining is mainly nuclear and regionalized at the caudal end of PD, suggesting that AR are active in the PD cells. The present results demonstrate variations in the regionalization and immunostaining pattern of AR during sexual maturity, probably in relation to the contribution of androgens reaching the different pituitary zones through blood vessels. Thus, the nuclear AR expression in pituitary PD of viscacha varies according to gonadal development and testosterone serum concentration. Our results are in agreement with those found by Wood and Newman [24], suggesting that a subset of unoccupied receptors is located in the cell cytoplasm in the absence of ligand.

Bibliographic reports referring to seasonal variations of RA are scarce. Lu et al. [56] have recently demonstrated a stronger expression of AR in scented glands during the breeding season, suggesting that androgens may directly influence the glandular function of the muskrats and courtship behavior. The viscacha is a seasonal breeding rodent and previous studies have demonstrated that testosterone acts on gonadotrophs, whether directly or indirectly, regulating their activity in the pituitary gland [28]. Castrated viscacha models have been used to examine the withdrawal effects of circulating androgens on pituitary cells, mainly on the gonadotrophs, due to their involvement in the reproductive processes. In addition, castration resulted in a significant decrease in the percentage of cells expressing AR throughout the PD [37]. The present results demonstrate changes in the amount of pituitary AR-ir cells during the annual reproductive cycle of adult male viscacha, which might be due to variations in the supply of ligands across blood irrigation. The maximum values of AR expression were found in the reproductive period and the minimum values in the regression period in relation to androgen concentrations. Thus, AR might regulate the activity of the pituitary cells involved in the reproductive cycle of adult male* Lagostomus*.

In conclusion, our results demonstrate that AR expression is related to the pituitary histophysiological changes during the growth and sexual maturity of male viscacha. Moreover, they are likely to participate in the seasonal reproduction control in adulthood. However, the precise action mechanism of AR in the pituitary viscacha needs further study.

## Figures and Tables

**Table 1:** AR expression in pituitary PD of male viscachas.

	Distribution	Immunostaining pattern	Morphological characteristics
MP-FETUS	Isolated or in small groups	Cytoplasmic and homogeneous	Oval or spherical cells

LP-FETUS	Isolated or in small groups	Cytoplasmic and homogeneous	Oval or spherical cells

Immature	Isolated or in small groups	Homogeneous and heterogeneous cytoplasm, some intensely stained nuclei	Cells oval in shape

Prepubertal	Isolated or in groups	Homogeneous cytoplasm, intensely nuclear staining	Irregular cells

Adult-Rep. P.	Isolated or in small groups	Nuclear immunoreactivity, scarce immunostained cytoplasms	Oval, spherical, or irregular cells

Adult-Reg. P.	Isolated	Nuclear immunoreactivity, scarce immunostained cytoplasms	Small and spherical cytoplasm

Adult-Rec. P.	Isolated or in small groups	Nuclear immunoreactivity, intense cytoplasmic staining	Spherical, oval, or irregular cytoplasms and nuclei, short cytoplasmic prolongation
